# Attention Distribution While Detecting Conflicts between Converging Objects: An Eye-Tracking Study

**DOI:** 10.3390/vision4030034

**Published:** 2020-07-22

**Authors:** Yke Bauke Eisma, Anouk E. Looijestijn, Joost C. F. de Winter

**Affiliations:** 1 Department of Cognitive Robotics, Faculty of Mechanical Engineering, Delft University of Technology, 2628 CD Delft, The Netherlands; y.b.eisma@tudelft.nl (Y.B.E.); anouk.looijestijn@gmail.com (A.E.L.); 2 Department of Control & Operations, Faculty of Aerospace Engineering, Delft University of Technology, 2628 CD Delft, The Netherlands

**Keywords:** conflict detection, eye-tracking, smooth pursuit

## Abstract

In many domains, including air traffic control, observers have to detect conflicts between moving objects. However, it is unclear what the effect of conflict angle is on observers’ conflict detection performance. In addition, it has been speculated that observers use specific viewing techniques while performing a conflict detection task, but evidence for this is lacking. In this study, participants (*N* = 35) observed two converging objects while their eyes were recorded. They were tasked to continuously indicate whether a conflict between the two objects was present. Independent variables were conflict angle (30, 100, 150 deg), update rate (discrete, continuous), and conflict occurrence. Results showed that 30 deg conflict angles yielded the best performance, and 100 deg conflict angles the worst. For 30 deg conflict angles, participants applied smooth pursuit while attending to the objects. In comparison, for 100 and especially 150 deg conflict angles, participants showed a high fixation rate and glances towards the conflict point. Finally, the continuous update rate was found to yield shorter fixation durations and better performance than the discrete update rate. In conclusion, shallow conflict angles yield the best performance, an effect that can be explained using basic perceptual heuristics, such as the ‘closer is first’ strategy. Displays should provide continuous rather than discrete update rates.

## 1. Introduction

In many types of occupations and daily activities, humans have to make decisions concerning spatial events that involve moving objects. A large number of empirical studies exist on this topic, for example in the area of car driving. These studies usually apply an egocentric perspective, where the observer performs temporal judgments while moving relative to one or more vehicles in the environment (e.g., [1,2]).

A less studied type of spatial task concerns the detection of a conflict between allocentric objects that move towards each other. This type of task has mainly been studied in air traffic control and other aviation contexts (e.g., [3,4]). As early as 1947, Gibson [5] described a variety of motion-picture-based tests for training and selection of air force personnel. One such test concerned the depiction of two animated planes, one overtaking the other. Before the overtaking point was reached, the planes disappeared behind a cloud, and the operator had to indicate at which imagined point the two planes would collide. Besides aviation, allocentric conflict detection tasks occur in areas such as gaming (e.g., robot combat or classical multidirectional shooter games; [6]) and monitoring of mobile agents. In the future, human operators may have to supervise the safe separation of drones [7], teleoperated cars [8], or mobile robots [9].

Human performance in allocentric conflict detection or arrival-time judgment tasks has also been studied in its own right without reference to a specific application (e.g., [10,11]). Kimball [12] argued that “time predictions about future positions of moving objects are made many times a day by virtually everyone” (p. 935). Much of the upcoming literature review pertains to air traffic control tasks, but we do not mean to imply that the application of our research is constrained to air traffic control.

### 1.1. The Effect of Conflict Angle on Conflict Detection Performance

Several studies have examined the accuracy with which operators detect whether two given aircraft are in conflict. Studies among university students [13] and licensed and trainee air traffic controllers [3] have found that participants are more likely to intervene when presented with a smaller conflict angle (45, 90, and 135/150 deg were used). These two studies also showed that participants frequently made false alarms, especially with smaller conflict angles. That is, with small conflict angles in particular, participants often indicated that there was a conflict when in fact there was a large minimum separation between the two aircraft.

Loft et al. [3] argued that air traffic controllers work under constraints of uncertainty: they have to estimate the aircraft trajectories and set criteria regarding whether to intervene. They noted that the effects of uncertainty are higher at smaller conflict angles because for small conflict angles, a small position estimation error can result in a large overlap of trajectories (see also [14], as cited in [15]). However, a potential confounder is that Loft et al. [3] defined a conflict as a loss of separation of 5 nautical miles, not a collision of two aircraft. Accordingly, a smaller conflict angle implies a longer period of violation of separation.

Pompanon and Raufaste [16] found, in a study among 556 novices who applied for a flight school, that 90 deg conflict angles resulted in shorter response times and higher accuracy in estimating the intersection point compared to smaller (45 deg) and higher (135 deg) conflict angles. These results appear to confirm the findings of Loft et al. [3] in that the small conflict angle of 45 deg yielded a large uncertainty/dispersion of the estimated intersection point. In a study among university students, Law et al. [11] found that moving objects on a parallel convergent (180 deg) trajectory yielded a higher conflict detection accuracy as compared to an oblique (45 deg) and perpendicular (90 deg) trajectory. They reported that “the effect of configuration seems to be primarily associated with visual scanning. As the objects are presented farther apart, accuracy decreases” [11] (p. 1188). However, eye movements were not examined in that study.

In summary, based on the above, it is unclear which type of conflict angle yields the best conflict detection performance, because results are contingent on various assumptions. Adding to the complication, in previous research, operators had to provide a ‘conflict’ or ‘no conflict’ response as quickly as possible, after which further responses were no longer possible [13,17]. This approach, in which only one data point per trial is obtained, cannot provide full insight into how operators accumulate evidence, or how they adjust their perceptual-cognitive strategies, as time elapses.

In a review on conflict detection, Xu and Rantanen [18] argued that operators might use various visual-cognitive processes during conflict detection tasks. For example, if the operator knows that the speeds of the convergent aircraft are equal, then the operator merely has to detect whether the distances of the two aircraft towards the conflict point are equal to infer that a conflict will occur. An alternative process would be to visually or cognitively extrapolate the motion of the aircraft [18]. Another model was proposed by Neal and Kwantes [13]. Their model assumes that operators iteratively sample evidence regarding the state of the world and accumulate it over time. They used their model to predict response times in a conflict decision task for different conflict angles but offered no further validations.

The visual-cognitive processes mentioned above seem plausible, but a weakness of the reviewed research is that the processes were not observed, but only inferred from performance measures. As pointed out by Xu and Rantanen [18], “the detection accuracy and the response time examined in the previous investigations seem to be the measures of the final product of conflict detection” (p. 3), not the actual process. Accordingly, the researchers recommended further research into operators’ conflict detection processes.

### 1.2. The Potential of Eye-Tracking in Conflict Detection Research

Eye-tracking can be used to unravel the relationships between the geometry of a scenario containing converging objects and operators’ visual-cognitive information processes. According to the strong eye-mind hypothesis [19], the location of observers’ eye fixations coincides with what the observer is mentally processing at that moment.

Thus far, only a few studies have examined how observers distribute their visual attention during allocentric conflict detection tasks. One relevant study is by Hunter and Parush [20], who recorded eye movements of university students observing two aircraft on a convergent trajectory. They found that the participants were more likely to scan between the two aircraft than towards the collision point. Based on this finding, they argued that “attention to the collision site may not be as essential to conflict detection as was previously thought” (p. 1732). However, important limitations are that Hunter and Parush’s [20] research was conducted with a relatively inaccurate head-mounted eye-tracker and that participants were presented with only one scenario. Furthermore, their analyses did not provide insight into how eye movement measures varied as the scenario progressed.

Another relevant study using eye-tracking was conducted by Pompanon and Raufaste [21]. In this work, 30 experienced air traffic controllers were asked to detect conflicts between two aircraft that flew on conflicting or divergent trajectories and at the same or different altitudes. Based on recorded first glances to areas of interest as well as response times, the authors proposed a model of human information processing. In short, this model asserted that operators first assess whether the aircraft converge or diverge. Next, they assess altitude differences between the two aircraft, and then they try to recognize geometric patterns in the trajectories and deduce whether the aircraft are in conflict. Pompanon and Raufaste’s [21] work is a good example of the usefulness of eye-tracking for this type of research. However, similar to Hunter and Parush [20], they did not show how the eye movements changed over time.

### 1.3. Study Aims

This study aimed to examine the effect of conflict angle on operators’ performance in allocentric conflict detection tasks. The above literature suggests that conflicts involving small conflict angles are easiest to detect yet prone to false positives. However, these results can be explained by the size of the separation zone (typically 5 nautical miles) and not by conflict angle per se. Another limitation of the existing research is that in the majority of the studies, participants provided only a single response per trial. Various studies have forwarded hypotheses of the visual-cognitive strategies that observers use while performing allocentric conflict detection tasks. However, the use of such strategies cannot be validly derived from response times alone.

In our experiment, we varied conflict angles from small (30 deg) to intermediate (100 deg) and large (150 deg) and examined how observers distribute their attention between two moving objects on a convergent trajectory. Measurements of eye movements and conflict detection were made continuously during each trial. Because the literature provides no clear leads, we formulated no a-priori hypotheses regarding the effect of conflict angle. In addition to eye movements, we acquired measures of conflict detection performance and self-reported difficulty. These two complementary measures were thought to reflect the difficulty of the conflict detection task.

In this study, we offered an additional manipulation: stimulus update rate. That is, all stimuli were offered with continuous movements and with discrete movements. Current radar systems provide discrete information because the radar sweeps at a fixed rate. A literature review by Chen and Thropp [22] of 50 empirical studies about the effect of update rate (i.e., frame rate) showed that a reduction of update rate is associated with a decrease of task performance. Therefore, we expected that performance in the conflict detection task would be better if the converging objects moved in a continuous as compared to a discrete manner. In Chen and Thropp’s [22] literature review, performance reductions were found in a variety of tasks, including placement, tracking, target recognition, and perceptual judgment tasks. For target recognition and perceptual judgments tasks, however, low update rates were sometimes found to yield a performance equivalent to baseline [22]. For example, a driving simulator study by Van Erp and Padmos [23] found no significant effect of update rate (3 up to 30 Hz were tested) on speed estimation accuracy. Accordingly, conflict detection performance may be unaffected by update rate.

## 2. Methods

### 2.1. Participants

Thirty-six persons participated in the experiment. They were students or recently graduated persons at the Delft University of Technology. The data of one participant were excluded because this participant did not perform the task as instructed. The remaining 35 participants consisted of 19 males and 16 females, between 18 and 31 years old (mean = 22.8, standard deviation (*SD*) = 2.91). Participants were offered compensation of 5 Euro for their time. This research was approved by the University’s Human Research Ethics Committee. A written informed consent form was signed by all participants before the start of the experiment.

### 2.2. Participants’ Task

**Figure 1:**
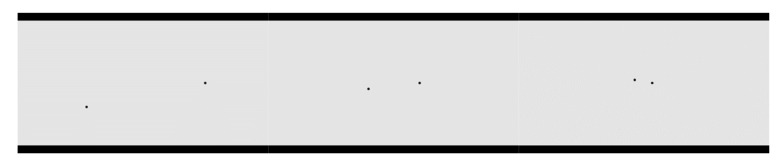
Screenshots of one scenario at three moments. **Left**: Beginning of the video, **Middle**: 10 s into the video, **Right**: 15 s into the video. This is a non-conflict scenario, with a conflict angle of 150 deg.

Participants watched a total of 36 videos, each containing a scenario of 20 s. In each scenario, two dots were linearly moving towards each other (Figure 1). Participants were instructed to keep the spacebar pressed when they thought the dots would collide.

After each scenario, participants indicated to what extent they agreed with the statement: “The task was difficult” on a scale from 0 (completely disagree) to 10 (completely agree). Next, the participant was shown his/her performance score for that scenario. The performance score was computed as the percentage of time that the spacebar was correctly pressed or released, depending on whether the scenario contained a conflict or no conflict, respectively.

Before the experiment, a calibration of the eye tracker was performed. Furthermore, participants were familiarized with the task using one training scenario with discrete stimuli. This scenario had a different geometry from the scenarios of the experiment. In the training scenario, a collision was presented. A break of a few minutes was held halfway during the experiment. The experiment lasted about 30 min per participant.

### 2.3. Apparatus

Eye movements were recorded at 2000 Hz using the SR-Research Eyelink 1000 Plus. Participants were asked to place their head in the head support. The stimuli were displayed on a 24 inch BENQ monitor with a resolution of 1920 × 1080 pixels (531 × 298 mm). The refresh rate of the monitor was 60 Hz. Based on an approximate distance of 91 cm between the monitor and the participant’s eyes, the monitor subtended viewing angles of 33 deg horizontally and 19 deg vertically.

### 2.4. Independent Variables

The first independent variable is the conflict angle between the two dots. In the literature, conflict angles have been divided into three categories: 0–60 deg (overtake), 60–120 deg (crossing), and 120–180 deg (head-on) [24]. For this experiment, one angle from each of these categories was used, namely 30, 100, and 150 deg.

The second independent variable was the update rate consisting of two levels: discrete and continuous. For the continuous stimuli, the update rate of the location of the dots was set equal to the video frame rate (30 frames per second). For the discrete stimuli, the update rate of the location of the dots was 2 times per second.

The third independent variable was the conflict outcome. In real-life tasks, objects have to retain a safe separation. For example, in air traffic control, aircraft have to be separated at least five nautical miles from each other (e.g., [25]). In this experiment, no separation zone was defined around the dots. The dots could either collide or not collide.

Each combination of independent variables was repeated three times in a different configuration, which meant that we rotated the entire stimulus with 0, 45, and 90 deg. In summary, participants were presented with 36 scenarios (3 conflict angles × 2 stimulus update rates × 2 conflict outcomes × 3 configurations). The sequence of the 36 scenarios was randomized for each participant.

### 2.5. Design of the Stimuli

The scenario consisted of a white (RGB: 0.9 0.9 0.9) background of 1920 × 960 pixels, on which two circular dots with a diameter of 18 pixels were shown (RGB: 0.1 0.1 0.1). The two dots (‘aircraft’) were moving at constant speeds and the same altitude on straight, converging courses [18].

Herein, we expressed the dimensions of the scenarios in pixels, as this information allows for exact reproduction of our methods. For our setup, a distance of 100 pixels on the screen corresponds to an angular range of approximately 1.7 deg. The speed of both dots was 26.4 pixels/s (528 pixels in 20 s or about 0.45 deg/s) during the entire experiment. For the discrete stimuli, the dots jumped forward 13.2 pixels per frame. This distance amounts to a change in visual angle of about 0.2 deg, which means that participants could keep a jump of a dot within foveal vision without re-fixating.

Dot 1 always started 480 pixels from the center of the screen. Dot 1 moved through the middle and ended 48 pixels from the mid-point. The heading of Dot 2 was determined by the conflict angle (i.e., 30, 100, or 150 deg) relative to Dot 1. For scenarios in which the dots collided, Dot 2 started 480 pixels from the center of the screen and ended 48 pixels from the midpoint, just as Dot 1. Thus, the collision occurred 18.3 s into the 20 s scenario.

For non-conflict scenarios, Dot 2 started with a 58-pixel offset so that the closest point of approach with respect to Dot 1 was 58 pixels, occurring 18.3 s into the scenario. This closest point of approach was determined using pilot tests. We ensured that the conflict detection task was not too easy (which would be when participants could easily see that no conflict would occur, e.g., at the beginning of the scenario) and not too difficult (i.e., which would be when participants could distinguish conflict from no conflict only during the last few seconds of the scenario).

All dimensions, including the closest point of approach of 58 pixels, were dimensionless. Participants were not provided with any reference about a numeric distance of speed and were therefore unable to interpret the task in reference to particular standards for safe separation.

An overview of the scenarios is shown in Table 1. Scenarios 19–36 are identical to Scenarios 1–18, but with discrete instead of continuous stimuli.

### 2.6. Dependent Variables

A median filter with a 100 ms interval was used to smoothen the raw eye-tracking data. When no eye data were available (e.g., during a blink), linear interpolation was used. The dependent variables were defined as follows:

Performance score (%): The performance score was computed as the percentage of time the participant had the spacebar correctly pressed or not pressed. For example, if a participant held the spacebar pressed between 7 and 12 s during a non-conflict scenario, the performance score for that participant in that scenario was (20 − 5 s)/20 s·100% = 75%.

Self-reported difficulty (0–10): A difficulty score between 0 and 10 was provided by the participants after each scenario, on a scale from (completely disagree) to 10 (completely agree).

Fixation rate (Hz): A higher fixation rate means that participants sample more elements from the scenario per time unit. For calculating the fixation rate, the eye-tracking data were partitioned into saccades and fixations in the same way as in Eisma, Cabrall, and De Winter [26]. First, the gaze speed was filtered with a Savitzky-Golay filter with order 2 and a frame length of 41. A saccade velocity threshold of 2000 pixels per second was used. The minimum fixation duration was set at 40 ms.

Mean fixation duration (s): During fixations, participants acquire information from the visual array. This measure is inversely related to the fixation rate. A longer mean fixation duration means that participants focused longer on the same element of the scenario.

Mean saccade amplitude (pixels): Saccade amplitude is another common measure in eye-tracking research [27]. A higher mean saccade amplitude indicates that participants have a broader spread of fixations.

Mean fixation amplitude (pixels): Smooth pursuit is a type of eye movement that involves the continuous movement of the eyes while tracking a moving object. From a visual inspection of participants’ *x* and *y* gaze coordinates, it became apparent that some fixations contained smooth pursuit, where participants followed one of the two dots. According to Holmqvist et al. [28], smooth pursuit is not easily identified, and “it is currently an open research problem to develop a robust and generic algorithm for such a purpose” (p. 152). Holmqvist et al. [28] also explained that standard velocity algorithms typically assign smooth pursuit data in the same category as fixations. Indeed, we observed that some fixations had a large amplitude, that is, the eyes traveled on the screen but without rapid saccade. Herein, we used the following measure of the degree of smooth pursuit: “as with saccades, the amplitude of smooth pursuit can also be calculated as the shortest distance between the points of on- and off-set” (p. 319). Holmqvist et al. [28] explained that this measure only works well when the direction of pursuit remains relatively constant, which we believe is a valid assumption in our case because the dots moved linearly. In summary, for each fixation, the straight-line distance from the start to the end moment of the fixation was computed and used as an index of the amount of pursuit. Thus, we did not classify fixations into smooth pursuit and no smooth pursuit but calculated the amplitude for each fixation.

Gaze coordinates on area of interest (AOI) (%): In accordance with Hunter and Parush [20], we assessed whether participants focused on the conflict point or one of the two dots. More specifically, we calculated the percentage of time that participants’ gaze coordinates were on one of the two dots within a radius of 100 pixels, hereafter referred to as the ‘dots AOI’. Additionally, we calculated the percentage of time that participants’ gaze coordinates were on the conflict point within a radius of 100 pixels, hereafter referred to as the ‘CP AOI’. In the case of non-conflict scenarios, the conflict point was defined as the mean of the coordinates of Dots 1 and 2 at their closest point of approach.

### 2.7. Statistical Analyses

First, scores on the dependent variables were compared between the scenarios with continuous and discrete update rates. Because we wanted to assess the main effect of update rate, paired-samples *t*-tests were used. Additionally, a three-way repeated-measures analysis of variance (ANOVA) of the fixation rate was performed to examine the effects of update rate (continuous versus discrete), conflict angle (30, 100, 150 deg), and conflict occurrence (no conflict versus conflict). Based on the small interaction effects with update rate (continuous versus discrete), we decided to aggregate the results of the continuous and discrete stimuli in subsequent analyses. Differences between the three conflict angles were compared using a repeated-measures ANOVA. Pairs of conflict angles were compared using paired-samples *t*-tests. *p*-values smaller than 0.05 were considered significant. Effect sizes between conditions were expressed as Cohen’s *d* and Cohen’s *d_z_*. Cohen’s *d_z_* describes the within-subjects effect size [29].

## 3. Results

### 3.1. Continuous Versus Discrete Stimuli

Table 2 shows that participants had a significantly higher performance score for continuous stimuli as compared to discrete ones. Furthermore, participants had a lower fixation rate and higher mean fixation duration for discrete stimuli as compared to the continuous stimuli. The effects for mean saccade amplitude, mean fixation amplitude, and self-reported difficulty were not statistically significant between continuous and discrete stimuli.

A three-way repeated measures full-factorial ANOVA for the fixation rate showed a significant effect of update rate (*F*(1,34) = 44.3, *p* < 0.001, η_p_^2^ = 0.57), conflict angle (*F*(2,68) = 267.7, *p* < 0.001, η_p_^2^ = 0.89), and conflict occurrence (*F*(1,34) = 18.0, *p* < 0.001, η_p_^2^ = 0.35).

**Figure 2:**
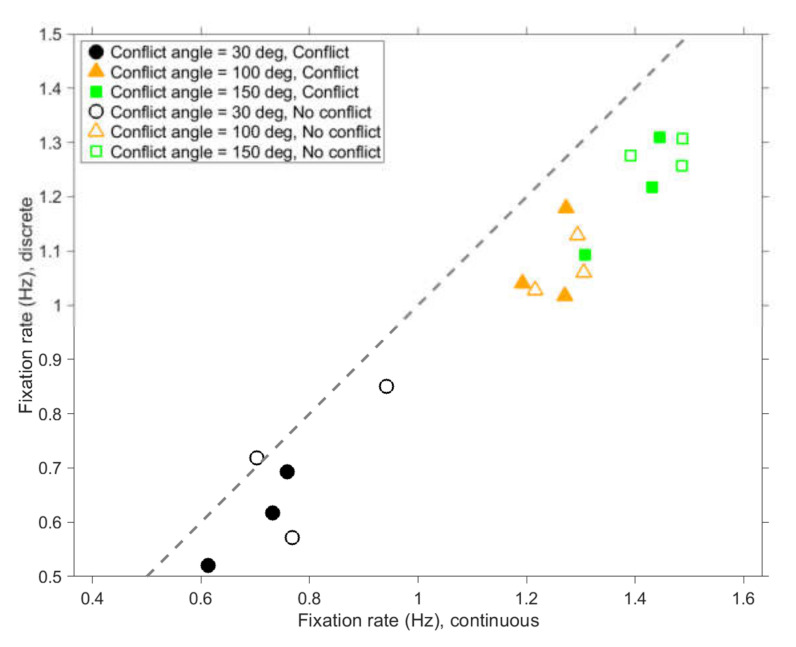
Mean number of fixations per second for scenarios with discrete stimuli versus scenarios with continuous stimuli. Each marker represents the average of 35 participants. The dashed line is the line of equality.

The interaction effect for update rate × conflict angle was small but significant (*F*(2,68) = 5.35, *p* = 0.007, η_p_^2^ = 0.14). Paired *t*-tests were conducted to examine the effect of update rate per conflict angle. For conflict trials, the effect of update rate increased with increasing conflict angle: *t*(34) = 3.42, 4.13, and 5.07, and *p* = 0.002, *p* < 0.001, and *p* < 0.001, for conflict angles of 30, 100, and 150 deg, respectively. A similar trend was observed for non-conflict trials: *t*(34) = 2.81, 4.93, and 4.01, and *p* = 0.008, *p* < 0.001, and *p* < 0.001, for conflict angles of 30, 100, and 15 deg, respectively. This interaction effect may be due to the fact that larger conflict angles involved a higher number of fixations (see Figure 2). The interaction effect for update rate × conflict occurrence was not significant (*F*(1,34) = 0.05, *p* = 0.825, η_p_^2^ = 0.00). Because the interaction effects with update rate were small, we averaged the results for the continuous and discrete stimuli in subsequent analyses.

### 3.2. Effect of Conflict Angle on Conflict Detection Performance

**Figure 3:**
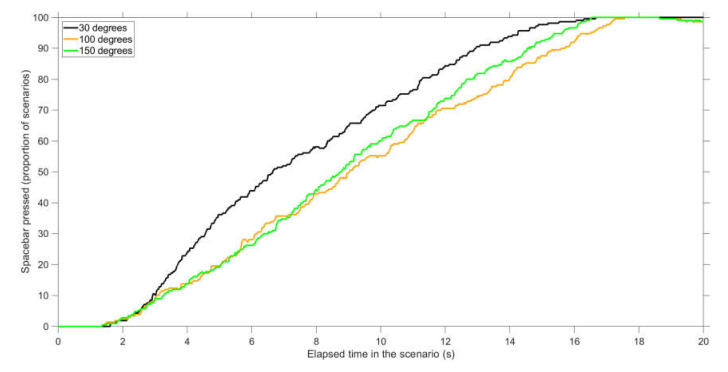
Percentage of participants who pressed the spacebar at that point in time during the scenario, for conflict scenarios. The proportion is calculated for 210 scenarios (35 participants × 6 scenarios per conflict angle).

Conflict scenarios: An evaluation of the spacebar pressings shows that conflicts were detected earlier for 30 deg conflict angles as compared to 100 and 150 deg conflict angles (Figure 3). For example, 5 s into the scenario, 36% of participants had pressed the spacebar in 30 deg scenarios, compared to 20% and 19% of participants in 100 and 150 deg scenarios, respectively. A repeated-measures ANOVA of the performance scores also showed a significant difference between conflict angles, *F*(2,68) = 12.2, *p* < 0.001. Paired *t*-tests showed significant differences between 30 and 100 deg scenarios (*t*(34) = 5.05, *p* < 0.001, *d* = 0.61, *d_z_* = 0.85), between 30 and 150 deg scenarios (*t*(34) = 3.60, *p* = 0.001, *d* = 0.54, *d_z_* = 0.61), but not between 100 and 150 deg scenarios (*t*(34) = −0.92, *p* = 0.364, *d* = −0.12, *d_z_* = −0.16).

**Figure 4:**
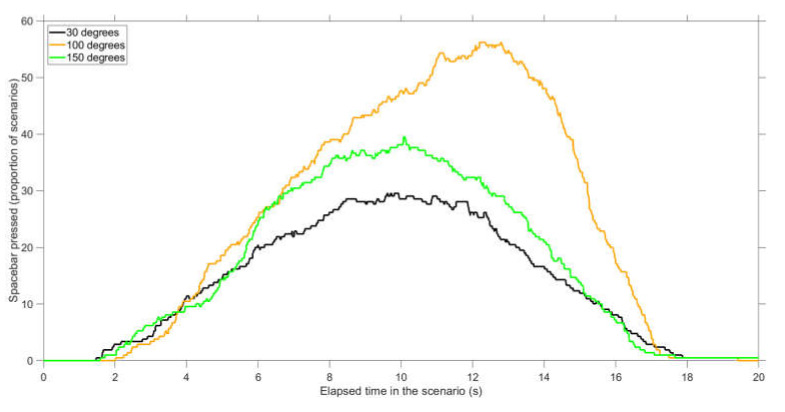
Percentage of participants who pressed the spacebar at that point in time during the scenario, for non-conflict scenarios. The proportion is calculated for 210 scenarios (35 participants × 6 scenarios per conflict angle).

Non-conflict scenarios: Furthermore, with 100 deg conflict angles, many participants falsely believed that there would be a conflict (Figure 4). The percentage of participants who falsely reported a conflict at a particular moment during the scenario was maximally 30%, 56%, and 40% for 30 deg (at 9.61 s), 100 deg (at 12.20 s), and 150 deg (at 10.07 s) conflict angles, respectively (see Figure 4 for a visualization). A repeated-measures ANOVA of the performance scores showed a significant difference between conflict angles, *F*(2,68) = 10.5, *p* < 0.001. Paired *t*-tests showed significant differences between 30 and 100 deg scenarios (*t*(34) = 5.17, *p* < 0.001, *d* = 0.97, *d_z_* = 0.87), between 100 and 150 deg scenarios (*t*(34) = −3.21, *p* = 0.003, *d* = −0.62, *d_z_* = −0.54), but not between 30 and 150 deg scenarios (*t*(34) = 1.11, *p* = 0.274, *d* = 0.27, *d_z_* = 0.19).

**Figure 5:**
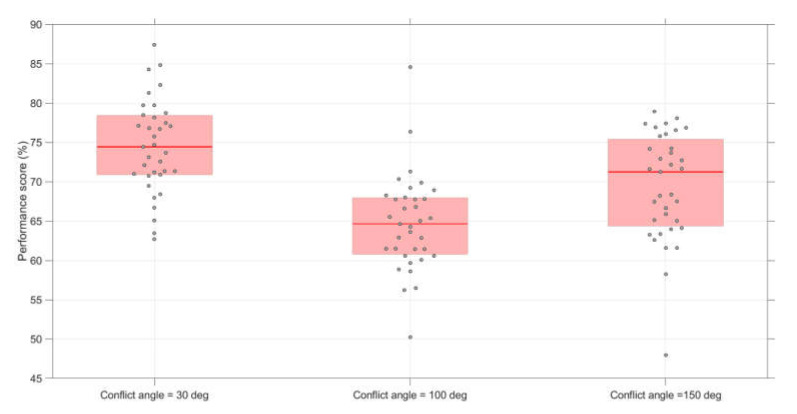
Boxplots of the performance scores per conflict angle. The score for each participant represents the average of 12 scenarios (conflict scenarios and non-conflict scenarios combined).

Conflict and non-conflict scenarios combined: Figure 5 shows that 30 deg conflict angles yielded the highest performance, and 100 deg conflict angles the lowest. A repeated-measures ANOVA of the performance scores showed a significant difference in performance between conflict angles, *F*(2,68) = 25.3, *p* < 0.001, η_p_^2^ = 0.43. Paired *t*-tests showed significant differences between 30 and 100 deg scenarios (*t*(34) = 8.21, *p* < 0.001, *d* = 1.61, *d_z_* = 1.39), between 30 and 150 deg scenarios (*t*(34) = 3.41, *p* = 0.002, *d* = 0.79, *d_z_* = 0.58), and between 100 and 150 deg scenarios (*t*(34) = −3.29, *p* = 0.002, *d* = −0.72, *d_z_* = −0.56).

### 3.3. Effect of Conflict Angle on Self-Reported Difficulty (Conflict and Non-Conflict Scenarios Combined)

**Figure 6:**
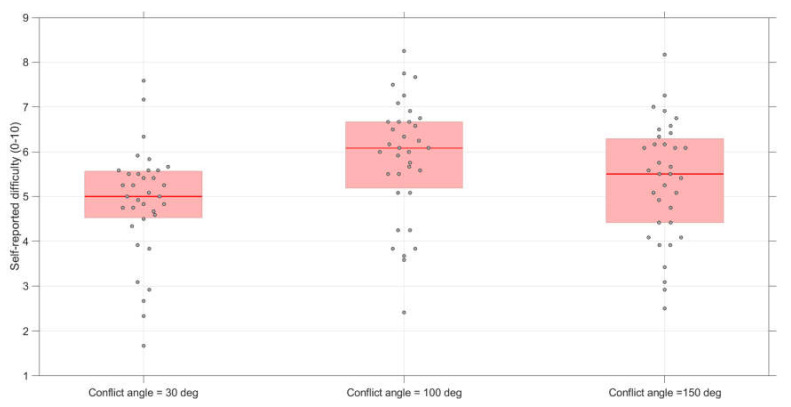
Boxplots of the self-reported difficulty scores per conflict angle. The score for each participant represents the average of 12 scenarios (conflict scenarios and non-conflict scenarios combined).

The self-reported difficulty was higher for the 100 deg conflict angle as compared to the other two conflict angles (Figure 6). A repeated-measures ANOVA showed significant differences between the three angles, *F*(2,68) = 30.1, *p* < 0.001, η_p_^2^ = 0.47. Paired *t*-tests further showed significant differences between 30 and 100 deg (*t*(34) = −7.61, *p* < 0.001, *d* = −0.77, *d_z_* = −1.29), between 30 and 150 deg (*t*(34) = −3.42, *p* = 0.002, *d* = −0.38, *d_z_* = −0.58), and between 100 and 150 deg (*t*(34) = 4.71, *p* < 0.001, *d* = 0.37, *d_z_* = 0.80).

### 3.4. Effect of Conflict Angle on Eye Movements (Conflict and Non-Conflict Scenarios Combined)

**Figure 7:**
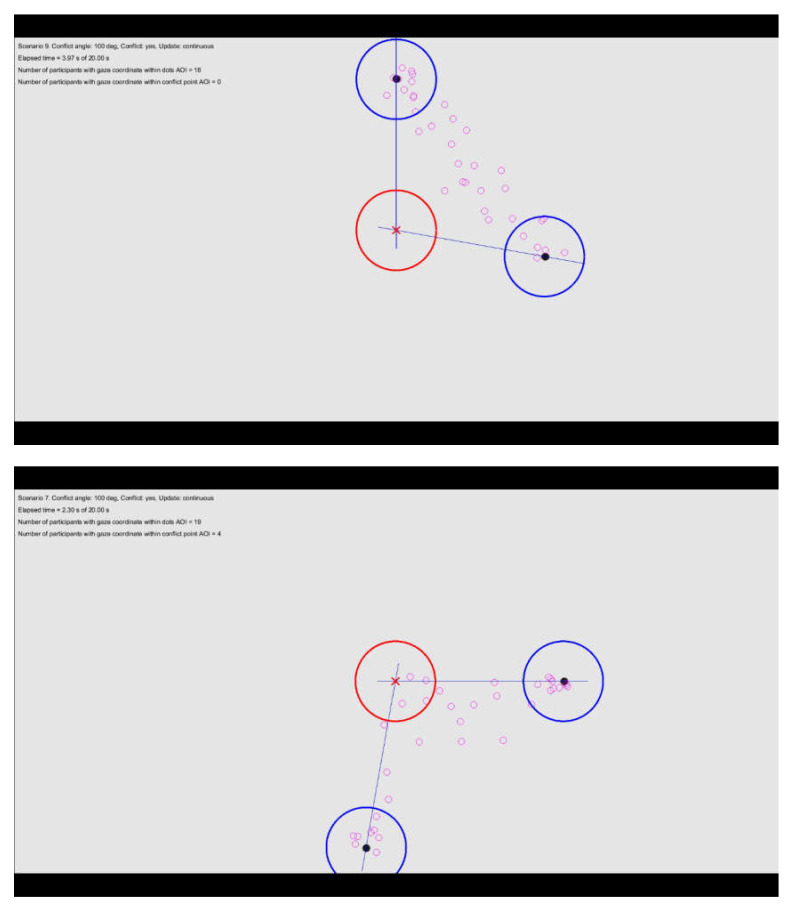
Snapshot from two selected scenarios (**Top**: Scenario 9, **Bottom**: Scenario 7) showing the dots (black circles), the gaze coordinates for the participants (*N* = 35), the conflict point (red X), the dots areas of interest (dots AOI, blue circles), and the conflict point area of interest (CP AOI, red circle). In the videos shown to the participants, only the two dots were visible.

Videos showing the gaze coordinates for all scenarios are available in the online data archive. From an inspection of the videos, we noted that participants predominantly looked at the dots (dots AOI) or in between the dots (i.e., close to an imaginary line connecting the two dots). Figure 7 (top) provides a video snapshot, illustrating that the participants sampled in between the dots or directly at the dots. However, in the 100 deg and 150 deg scenarios, participants sometimes directed their gaze towards the conflict point angles (see Figure 7, bottom, for an illustration for looking towards the conflict point).

**Figure 8:**
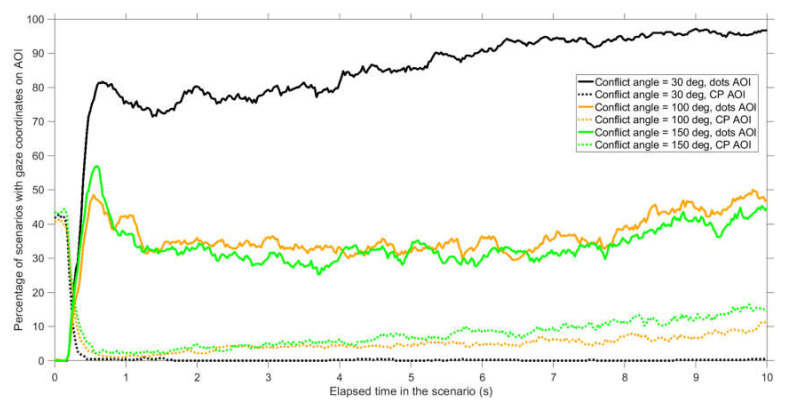
Percentage of participants with gaze coordinates in an area of interest (AOI) as a function of elapsed time in the scenario. A distinction is made between AOIs surrounding the dots (dots AOI) and the AOI surrounding the conflict point (CP AOI). The shown values represent averages for 35 participants and 12 scenarios per participant (conflict scenarios and non-conflict scenarios combined). For example, at an elapsed time of 4 s, for scenarios with 30 deg conflict angle, participants looked at the dots AOI in 342 of the 420 cases (81.4%), and at the CP AOI in only 1 of the 420 cases (0.2%). Only the first 10 s of the scenario are shown because from 10 s onwards, the AOIs started to overlap.

Figure 8 provides further information about participants’ looking behavior at AOIs as a function of elapsed time in the scenario. It can be seen that for 30 deg conflict angles, participants predominantly looked at the dots AOI and hardly looked at the conflict point (CP AOI). For 100 deg conflict angles, and especially for 150 deg conflict angles, participants did look at the conflict point to some extent. Most of the remaining time was spent looking in between the dots (see also Figure 7).

**Figure 9:**
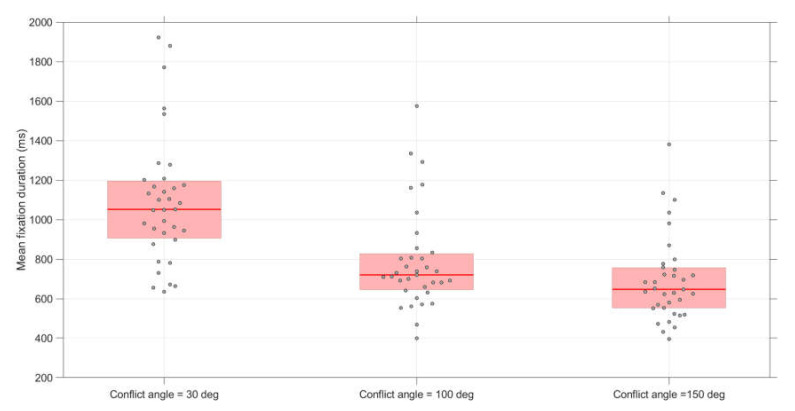
Boxplots of the mean fixation duration per conflict angle. The score for each participant represents the average of 12 scenarios (conflict scenarios and non-conflict scenarios combined).

As shown in Figure 9, mean fixation durations were longer for smaller conflict angles. A repeated-measures ANOVA showed significant effects of conflict angle, *F*(2,68) = 68.7, *p* < 0.001, η_p_^2^ = 0.72. Paired *t*-tests also showed significant differences in fixation duration between 30 and 100 deg (*t*(34) = 8.27, *p* < 0.001, *d* = 1.05, *d_z_* = 1.40), between 30 and 150 deg (*t*(34) = 11.10, *p* < 0.001, *d* = 1.46, *d_z_* = 1.88), and between 100 and 150 deg (*t*(34) = 4.76, *p* < 0.001, *d* = 0.41, *d_z_* = 0.80).

**Figure 10:**
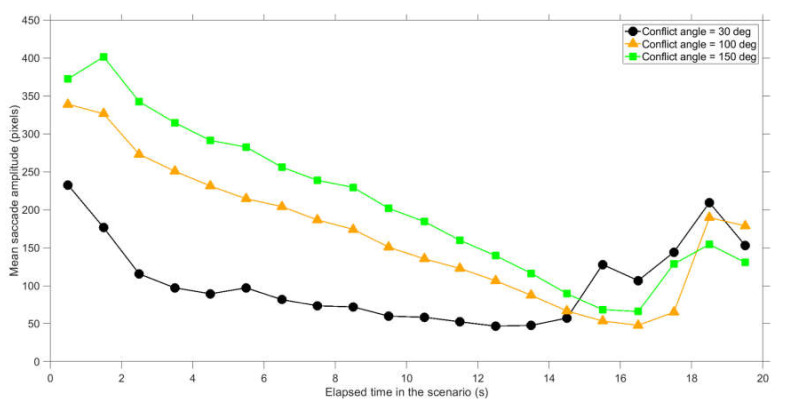
Mean saccade amplitude per conflict angle, where the end moments of saccades are divided into 1 s bins since the start of the scenario. The shown values represent averages for 35 participants and 12 scenarios per participant (conflict scenarios and non-conflict scenarios combined).

**Figure 11:**
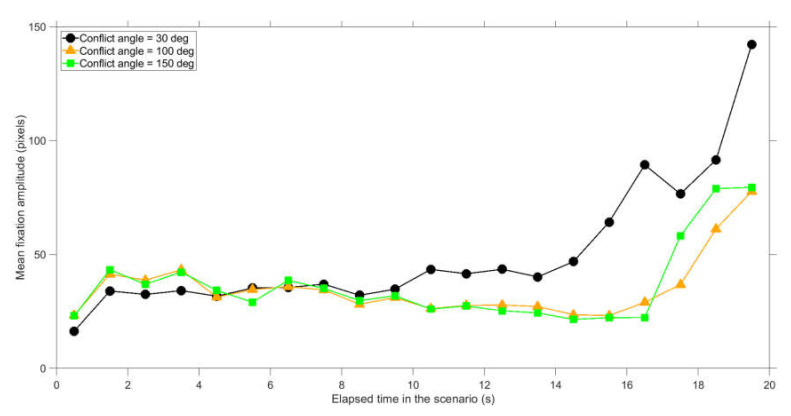
Mean fixation amplitude per conflict angle, where the end moments of saccades are divided into 1 s bins since the start of the scenario. The shown values represent averages for 35 participants and 12 scenarios per participant (conflict scenarios and non-conflict scenarios combined).

Further analysis of the data revealed dynamic viewing patterns as a function of elapsed time during the scenario. The saccade amplitude showed interpretable patterns: saccades had a larger amplitude earlier in the scenario as well as for larger conflict angles (Figure 10). This decrease of amplitude can be explained by the fact that the distance between the dots linearly decreases with elapsed time. At 18.3 s in the scenario, the two dots collided. When the outcome of the scenario (i.e., collision or no collision) becomes evident, participants sometimes sample elsewhere on the screen, which can explain the increase of saccade amplitude near the end of the scenario. The fixation amplitude describes whether participants tracked an object using pursuit movement. The fixation amplitude also increased near the end of the scenario, especially for the small conflict angle of 30 deg (Figure 11).

### 3.5. Scenario-Specific Effects

An overview of the dependent measures for each of the 18 scenarios is provided in the Supplementary Materials (Table S1). For most scenarios, participants distributed their attention towards Dot 1 and 2 in an approximately 50–50% manner. However, for some scenarios, participants focused more on one of the dots. In particular, if most (>70%) of the attention went to one of the two dots, this pertained to a dot that was moving horizontally or downward.

**Figure 12:**
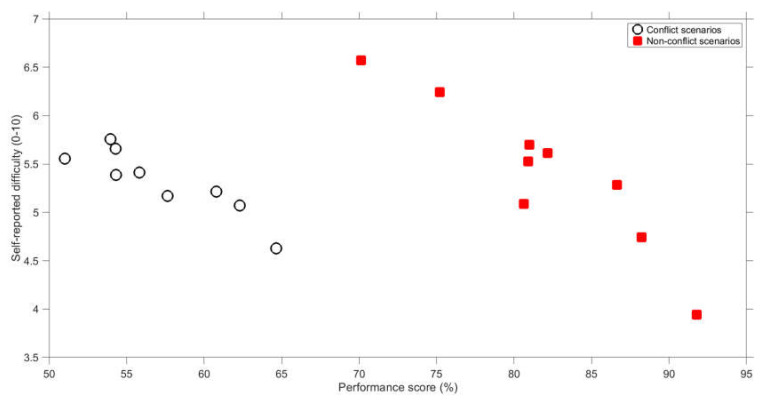
Mean self-reported difficulty score versus mean performance score for conflict scenarios and non-conflict scenarios. Each marker represents the average of 35 participants and 2 scenarios (discrete and continuous scenarios are combined).

Another noteworthy finding is that, at the level of scenarios, better performance was associated with a lower self-reported difficulty (Table S1). This relationship, which is shown in Figure 12, held for conflict scenarios (*r* = −0.89, *n* = 9) and non-conflict scenarios (*r* = −0.93, *n* = 9). In other words, participants were able to reliably assess which scenarios are more difficult than others.

## 4. Discussion

This study aimed to investigate the effects of conflict angle on eye movements in an allocentric conflict detection task. Additionally, we studied the effects of discrete versus continuous screen-update rates on eye movements and conflict detection performance.

### 4.1. Effects of Conflict Angle

The results showed that conflict detection is a dynamic task in which participants’ judgments become more accurate as the time to conflict decreases (Figure 3 and Figure 4, see also Supplementary Figure S1). These findings serve as support for Neal and Kwantes [13], who argued that observers accumulate evidence over time until reaching a decision threshold.

The results showed that conflict angles of 30 deg yielded better performance and lower ratings of task difficulty than 150 deg conflict angles. In turn, 100 deg conflict angles yielded the lowest performance and were deemed the most difficult. For 30 deg angles, if there was a conflict, participants detected that conflict early, and if there was no conflict, participants were unlikely to indicate that there was. Thus, the high-performance score for the 30 deg conflict angle was because of both improved hits and reduced false positives. Figure S1 in the Supplementary Materials provides support for these observations using an index of perceptual sensitivity (*d*′), calculated using detection theory [30]. Results for the response bias (β), also shown in the Supplementary Materials (Figure S2), indicate that participants behaved approximately as an ideal observer, that is, they assigned equal weight to Type II errors (failing to report a conflict in conflict trials) and Type I errors (reporting a conflict in non-conflict trials).

How can the superior performance for 30 deg conflict angles be explained, and why did 100 deg conflict angles yield the poorest performance? Gilden [31] argued that participants often use simple kinematic heuristics when gaining awareness of dynamical systems. The results of our study can also be explained with kinematic heuristics. For 30 deg conflict angles, it may be easy for participants to detect an imminent collision, because if one dot travels behind the other at a fixed speed, then the observer knows that the dots will not collide [11,18,21]. If the time in the scenario elapses, the relative distance of the dots to the conflict point keeps increasing, so it should become more and more evident to the observer that the trailing dot will not overtake the leading dot (see Supplementary Figure S3 for a relative distance graph). Tresilian [2] explained that this “closer is first” (p. 240) rule is easiest to apply when the two targets move in parallel. For 150 deg angles, a conflict may also be easy to detect, for example as an offset from an imaginary line connected the two converging dots [21]. For 100 deg conflict angles, however, there may have been no such kinematic rules that the participants could apply.

For 30 deg conflict angles in particular, participants employed smooth pursuit eye movements while not glancing at the future conflict point. These patterns are in agreement with the ‘closer is first’ strategy. By tracking the two dots, it may become apparent whether the dots move at a constant velocity and side-by-side (resulting in a collision) or that one dot lags behind the other (resulting in a safe pass). We also observed that participants preferred to look most at a dot that was moving downward or horizontally (see Section 3.5), which is consistent with literature about pursuit movements [32].

For larger conflict angles (100 and 150 deg), participants showed a higher number of fixations, and the gaze coordinates were often in between the dots and the conflict point AOIs. For these conflict angles, the dots are further apart on the screen, and observers cannot apply smooth pursuit of one dot while keeping the other dot within the foveal region. The phenomenon of looking at the conflict point can be explained by required eye-movement effort, in line with Wickens’ [33] Saliency-Effort-Expectancy-Value (SEEV) model: For 150 deg conflict angles, the conflict point lies in between the two dots, making it less effort for participants to sample towards that conflict point, as compared to smaller conflict angles. In summary, the eye-movement patterns are explainable in terms of the distance between the dots, which is larger when the conflict angle is higher.

### 4.2. Effects of Update Rate

Continuous stimuli yielded a statistically significant improvement of conflict detection performance score as compared to discrete stimuli, with a Cohen’s *d* effect size of 0.46. In other types of tasks, such as driving in a virtual driving simulator, considerably stronger effects of visual update rate have been observed. For example, Van Erp and Padmos [23] observed a factor 3 difference in lane-keeping performance between low (3 Hz) and normal (30 Hz) update rate conditions. The relatively small effects of update rate in the present study can be explained by the fact that the current task was an open-loop task in which participants did not rely on feedback to respond. The discrete presentation resulted in a delayed perception, where participants had to wait for a movement of the dot by keeping it in foveal vision in order to determine its velocity. In a closed-loop task such as car driving, the effect of a limited update rate would cause not only a delay in perception but also a delayed steering response, resulting in reduced stability of control [22].

A strong effect of update rate was found for fixation duration. That is, with discrete stimulus movement, observers fixated longer, and exhibited fewer fixations per second, as compared to continuous stimulus movement. This difference may have occurred because, with a discrete presentation of stimuli, it takes time to extract heading information. The increase of fixation duration can be interpreted as indicative of increased processing load and difficulty of interpreting the stimuli [27,34]. It is noted that in our experiment, information about the speed of the dots had to be obtained from the movement of the dots, whereas in actual applications, speed and heading information may also be available in an accompanying text label. Regardless, our results suggest that (radar) displays should update continuously rather than intermittently.

### 4.3. Limitations

Our task was simple, comprising of two moving dots moving at the same altitude, and a limited number of geometries of the scenarios. It is still to be determined how passing in front or behind, distance to the closest point of approach, relative speed, and the number of objects would affect attention distribution. If there are multiple moving objects, visual search for conflicts may become a crucial factor. A conflict between two moving targets may be hard to identify among multiple other moving targets, especially if the targets are far apart [15,35]. In real air traffic control, aircraft are accompanied by flight labels. The altitude labels are an important source for determining whether aircraft are in conflict [21].

Real air traffic control tasks involve multitasking, such as communication and teamwork, which in turn affect eye movements [36,37,38]. Furthermore, it is known that air traffic control operators tend to experience their task as safety-critical and sometimes stressful [39,40]. In our study, participants assigned about equal weight to false negatives and false positives (see Figure S2 in the Supplementary Materials). It is expected that in real air traffic control, operators are more likely to prevent false negatives (i.e., apply a cautious strategy).

This research was conducted with engineering students. Because we tested fundamental perceptual principles, we believe that our findings are generalizable to other participant groups. However, some differences between experts and novices are to be expected. Loft et al. [3] found that air traffic control experts were more likely to intervene than trainees. Similarly, Bisseret [41] argued that experienced operators swiftly respond to a conflict, whereas trainees may feel hesitant to act once they detect a conflict. Van Meeuwen et al. [42] found that, for a task in which participants had to provide the optimal order of arrival of aircraft, expert air traffic controllers reached better solutions and applied more efficient visual scan paths as compared to novice air traffic controllers.

A final limitation is that our study was concerned with conflict detection only, with high performance in conflict trials being determined by pressing the spacebar as early as possible (Figure S4). Hilburn [43] argued that conflict resolution involves task demands that differ from conflict detection. For example, he commented that: “Similarly, head on situations seem easier to detect, but (because of high closure speed) are more difficult to resolve” (p. 57). Future research could focus on examining the interplay between conflict detection and conflict resolution.

## 5. Conclusions

It is concluded that conflict detection performance is better for small conflict angles (30 deg) than for near-perpendicular angles (100 deg). A small conflict angle results in pursuit movement, whereas larger conflict angles result in higher eye-movement activity and eye movements in between the dots rather than at the dots. Additionally, continuously moving stimuli yield better conflict detection performance than stimuli that moved in a discrete manner.

## Figures and Tables

**Table 1:** Characteristics of Scenarios 1–18.

Scenario Number	Conflict Angle (deg)	Dot 1 Heading (deg)	Dot 2 Heading (deg)	Dot 1 Coordinate (*x*, *y* in pixels)		Dot 2 Start Coordinate (*x*, *y* in pixels)		Conflict	Relative Distance to CP at Start (Dot 1/Dot 2)	Dot 2 Passing Dot 1
1	30	270	300	1440	480	1375	723	Yes	1	
2	30	225	195	1299	141	1081	15	Yes	1	
3	30	180	150	960	0	717	65	Yes	1	
4	30	270	240	1440	480	1431	225	No	0.89	Behind
5	30	225	195	1299	141	1113	−34	No	0.89	Behind
6	30	180	150	960	0	705	9	No	0.89	Behind
7	100	270	10	1440	480	874	955	Yes	1	
8	100	225	125	1299	141	563	205	Yes	1	
9	100	180	280	960	0	1435	566	Yes	1	
10	100	270	10	1440	480	840	909	No	1.08	In front
11	100	225	325	1299	141	1178	868	No	1.08	In front
12	100	180	280	960	0	1389	600	No	1.08	In front
13	150	270	120	1440	480	541	239	Yes	1	
14	150	225	75	1299	141	493	606	Yes	1	
15	150	180	330	960	0	1201	899	Yes	1	
16	150	270	60	1440	480	529	664	No	1.03	In front
17	150	225	75	1299	141	468	554	No	0.97	Behind
18	150	180	330	960	0	1144	911	No	1.03	In front

Note. Scenarios 1–18 were presented with continuous movements of the dots. Scenarios 19–36 are identical to Scenarios 1–18, but with discrete movement. The (0, 0) coordinate is the left top corner of the video. Heading angles of 0, 90, 180, and 270 deg are north, east, south, and west, respectively. CP = conflict point.

**Table 2:** Means and standard deviations (*SD*) of the dependent variables for continuous and discrete stimuli, as well as results of paired *t*-tests between the scores for continuous and discrete stimuli.

	Continuous Stimuli		Discrete Stimuli					
	Mean	*SD*	Mean	*SD*	*t*(34)	*p*	Cohen’s *d*	Cohen’s *d_z_*
Fixation rate (Hz)	1.145	0.298	0.993	0.280	6.66	<0.001	0.53	1.13
Mean fixation duration (ms)	813	235	905	269	−4.40	<0.001	−0.36	−0.74
Mean saccade amplitude (pixels)	182	31	179	33	1.47	0.151	0.10	0.25
Mean fixation amplitude (pixels)	36	12	35	13	0.83	0.411	0.08	0.14
Performance score (%)	70.8	5.59	68.3	5.56	2.09	0.044	0.46	0.35
Self-reported difficulty (0–10)	5.30	1.28	5.43	1.24	–1.31	0.198	−0.11	−0.22

Note. The results for each participant were averaged for the 18 continuous scenarios and the 18 discrete scenarios.

## Data Availability

Raw data, analysis scripts, and a video showing the participants’ gaze coordinates relative to the converging dots are available online at https://doi.org/10.4121/uuid:41914583-a126-4a24-9763-0dea6f15cd2d.

## Supplementary Materials

The following are available online at https://www.mdpi.com/2411-5150/4/3/34/s1, Figure S1: Perceptual sensitivity (d’) as a function of elapsed time during the scenario, calculated from the results shown in Figure 3 and Figure 4; Figure S2: Response bias (β) as a function of elapsed time, calculated from the results shown in Figure 3 (showing hit rates) and Figure 4 (showing false alarm rates); Figure S3: Ratio between the distance from Dot 1 to the conflict point and the distance from Dot 2 to the conflict point; Figure S4: Mean first moment of pressing the spacebar versus mean performance score for conflict scenarios; Table S1: Means (color-coded) and standard deviations of dependent variables per scenario (N = 35).
