# Supplementary material for: Attention Distribution While Detecting Conflicts between Converging Objects: An Eye-Tracking Study
Source: Vision (Basel). 2020 Jul 22;4(3):34. doi: 10.3390/vision4030034 (PMC7558710; doi:10.3390/vision4030034)
Supplement: Supplementary file 1 [file vision-04-00034-s001.pdf]

# Supplementary materials

Table S1. Means (color-coded) and standard deviations of dependent variables per scenario ($N = 35$).

| Scenario number | Conflict angle (deg) | Conflict | 1. Fixation rate (Hz) | | 2. Mean fixation duration (ms) | | 3. Mean saccade amplitude (pixels) | | 3. Mean fixation amplitude (pixels) | | Proportion of time on Dot 1 | | Proportion of time on dots AOI | | Proportion of time on CP AOI | | Performance score (%) | | Self-reported difficulty (0-10) | | Self-reported difficulty (0-10) | | Number of spacebar presses (#) | |
|---|---|---|---|---|---|---|---|---|---|---|---|---|---|---|---|---|---|---|---|---|---|---|---|---|
| 1 | 30 | Yes | 0.566 | 0.218 | 1194 | 452 | 126 | 38 | 39 | 15 | 0.750 | 0.139 | 0.951 | 0.039 | 0.262 | 0.050 | 60.772 | 15.909 | 5.214 | 1.655 | 5.214 | 1.655 | 1.01 | 0.08 |
| 2 | 30 | Yes | 0.674 | 0.289 | 1000 | 440 | 106 | 26 | 35 | 15 | 0.558 | 0.175 | 0.947 | 0.031 | 0.262 | 0.036 | 64.653 | 13.664 | 4.629 | 1.601 | 4.629 | 1.601 | 1.01 | 0.08 |
| 3 | 30 | Yes | 0.726 | 0.264 | 1041 | 479 | 112 | 30 | 38 | 17 | 0.732 | 0.132 | 0.932 | 0.063 | 0.275 | 0.042 | 62.305 | 15.904 | 5.071 | 1.520 | 5.071 | 1.520 | 1.01 | 0.08 |
| 4 | 30 | No | 0.670 | 0.301 | 1245 | 523 | 121 | 41 | 42 | 19 | 0.232 | 0.138 | 0.952 | 0.048 | 0.237 | 0.049 | 80.625 | 15.856 | 5.086 | 1.881 | 5.086 | 1.881 | 0.60 | 0.42 |
| 5 | 30 | No | 0.711 | 0.246 | 1111 | 473 | 155 | 65 | 39 | 14 | 0.503 | 0.160 | 0.946 | 0.025 | 0.250 | 0.035 | 86.654 | 14.155 | 5.286 | 2.136 | 5.286 | 2.136 | 0.44 | 0.40 |
| 6 | 30 | No | 0.896 | 0.365 | 977 | 359 | 110 | 23 | 40 | 17 | 0.636 | 0.163 | 0.937 | 0.052 | 0.283 | 0.046 | 91.792 | 11.124 | 3.943 | 1.748 | 3.943 | 1.748 | 0.26 | 0.33 |
| 7 | 100 | Yes | 1.116 | 0.306 | 783 | 259 | 187 | 44 | 32 | 15 | 0.707 | 0.090 | 0.711 | 0.123 | 0.399 | 0.094 | 50.999 | 19.775 | 5.557 | 2.068 | 5.557 | 2.068 | 1.11 | 0.27 |
| 8 | 100 | Yes | 1.226 | 0.365 | 723 | 268 | 215 | 45 | 38 | 20 | 0.625 | 0.111 | 0.739 | 0.131 | 0.335 | 0.048 | 55.816 | 18.447 | 5.414 | 1.873 | 5.414 | 1.873 | 1.07 | 0.18 |
| 9 | 100 | Yes | 1.144 | 0.388 | 788 | 365 | 191 | 52 | 36 | 17 | 0.529 | 0.128 | 0.671 | 0.153 | 0.345 | 0.052 | 53.956 | 17.928 | 5.757 | 1.729 | 5.757 | 1.729 | 1.03 | 0.12 |
| 10 | 100 | No | 1.211 | 0.330 | 752 | 215 | 184 | 46 | 28 | 13 | 0.655 | 0.133 | 0.687 | 0.124 | 0.401 | 0.093 | 70.112 | 14.323 | 6.571 | 1.582 | 6.571 | 1.582 | 0.86 | 0.38 |
| 11 | 100 | No | 1.121 | 0.373 | 878 | 395 | 176 | 42 | 36 | 15 | 0.742 | 0.117 | 0.732 | 0.131 | 0.318 | 0.056 | 82.180 | 16.101 | 5.614 | 1.922 | 5.614 | 1.922 | 0.57 | 0.39 |
| 12 | 100 | No | 1.183 | 0.379 | 806 | 270 | 187 | 48 | 37 | 17 | 0.604 | 0.129 | 0.680 | 0.150 | 0.314 | 0.059 | 75.206 | 15.799 | 6.243 | 1.804 | 6.243 | 1.804 | 0.76 | 0.46 |
| 13 | 150 | Yes | 1.324 | 0.305 | 648 | 178 | 243 | 59 | 30 | 17 | 0.555 | 0.125 | 0.659 | 0.147 | 0.458 | 0.114 | 54.295 | 14.676 | 5.657 | 1.748 | 5.657 | 1.748 | 1.09 | 0.23 |
| 14 | 150 | Yes | 1.378 | 0.348 | 638 | 203 | 228 | 49 | 34 | 22 | 0.665 | 0.099 | 0.685 | 0.151 | 0.445 | 0.096 | 57.644 | 17.003 | 5.171 | 1.761 | 5.171 | 1.761 | 1.04 | 0.19 |
| 15 | 150 | Yes | 1.200 | 0.381 | 735 | 304 | 219 | 53 | 39 | 20 | 0.629 | 0.107 | 0.645 | 0.148 | 0.459 | 0.090 | 54.298 | 16.902 | 5.386 | 1.871 | 5.386 | 1.871 | 1.07 | 0.18 |
| 16 | 150 | No | 1.334 | 0.366 | 735 | 278 | 237 | 50 | 35 | 21 | 0.582 | 0.121 | 0.670 | 0.145 | 0.478 | 0.133 | 88.244 | 12.186 | 4.743 | 1.550 | 4.743 | 1.550 | 0.47 | 0.42 |
| 17 | 150 | No | 1.397 | 0.426 | 690 | 307 | 233 | 50 | 32 | 17 | 0.636 | 0.120 | 0.705 | 0.142 | 0.451 | 0.107 | 80.997 | 16.776 | 5.700 | 1.733 | 5.700 | 1.733 | 0.56 | 0.43 |
| 18 | 150 | No | 1.371 | 0.390 | 711 | 320 | 218 | 52 | 35 | 20 | 0.669 | 0.092 | 0.634 | 0.147 | 0.442 | 0.088 | 80.906 | 17.119 | 5.529 | 1.863 | 5.529 | 1.863 | 0.61 | 0.46 |

*Note*. The results for each participant were averaged for the 18 continuous scenarios and the 18 discrete scenarios. The additional measure ‘proportion of time on Dot 1’ represents the proportion of time that the gaze coordinate was closer to Dot 1 than to Dot 2. CP = conflict point.

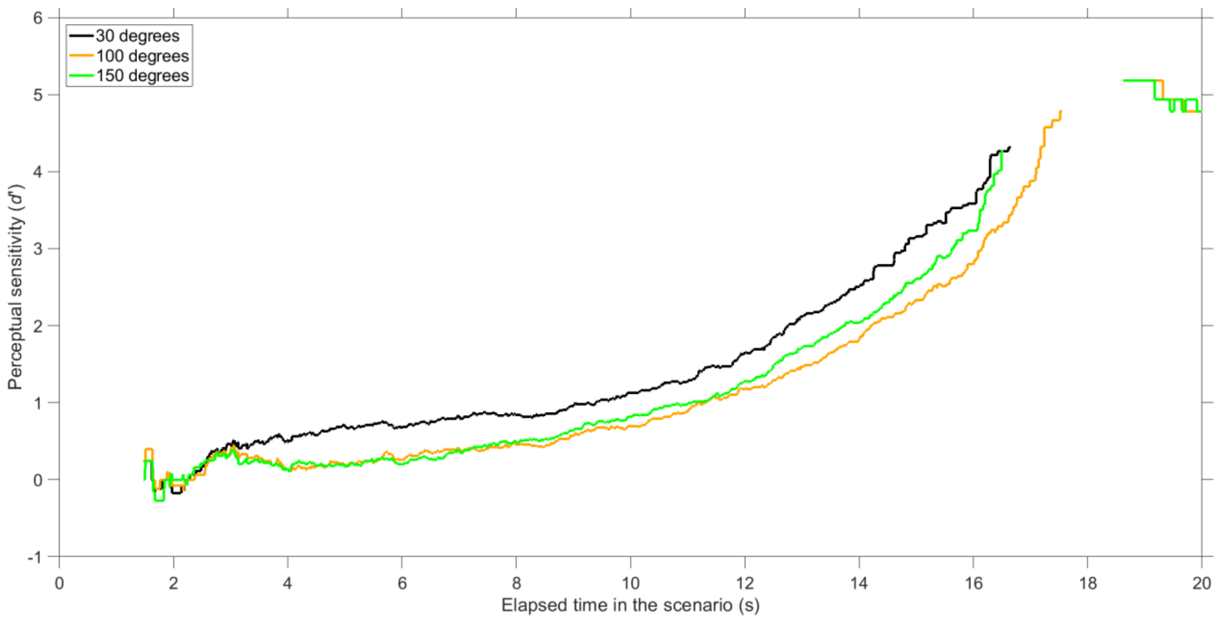


*Figure S1*. Perceptual sensitivity (*d'*) as a function of elapsed time during the scenario, calculated from the results shown in Figures 3 and 4. It can be seen that perceptual sensitivity is highest for 30 deg conflict angles. Also, perceptual sensitivity increases with elapsed time, which can be explained because it gradually becomes evident whether or not a collision will occur.

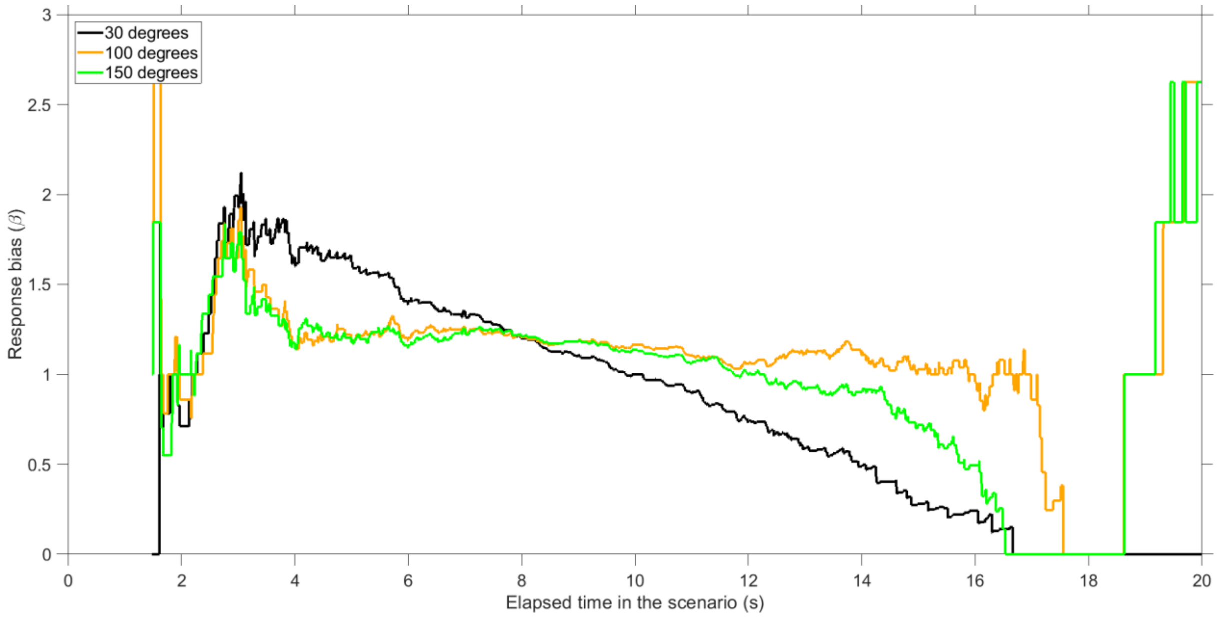


*Figure S2*. Response bias (β) as a function of elapsed time, calculated from the results shown in Figure 3 (showing hit rates) and Figure 4 (showing false alarm rates). β = 1 would represent an 'ideal observation' where the miss rate equals the false positive rate. It can be seen that β is about 1 for 100 deg and 150 deg conflict angles, whereas β decreases with elapsed time for 30 deg conflict angles. To illustrate, at about 16 seconds into the 30-deg scenarios, the miss rate was low (1%, or 99% hit rate) but the false alarm rate was high (8%), indicating that participants were cautious (i.e., liberal, low β) at that point in time. In other words, in non-conflict scenarios, some participants kept pressing the spacebar to indicate that the dots could collide even

when the dots would not collide, an effect that may be due to a delay in the human response.

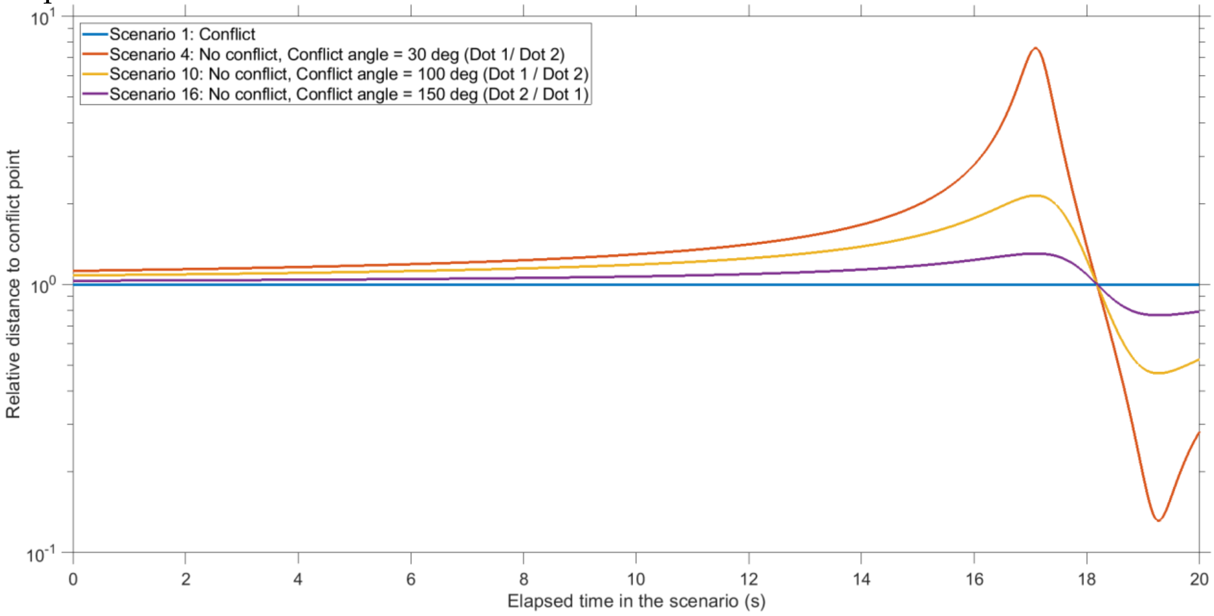


*Figure S3*. Ratio between the distance from Dot 1 to the conflict point and the distance from Dot 2 to the conflict point. For example, if the value equals 2, then one dot is twice as far from the conflict point as the other dot. Note that the closest point of approach is at 18.3 s.

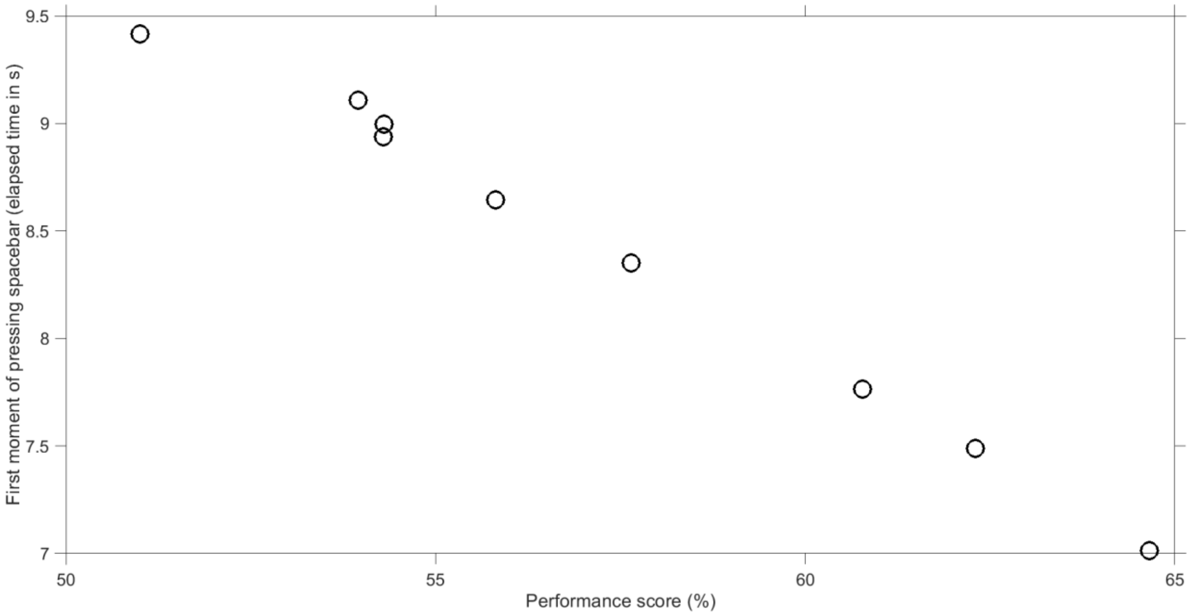


*Figure S4*. Mean first moment of pressing the spacebar versus mean performance score for conflict scenarios. Each marker represents the average of 35 participants and 2 scenarios (discrete and continuous scenarios are combined). The strong correlation indicates that the moment of pressing the spacebar and the performance score are redundant variables at the level of scenarios.
